# Predicting hair cortisol levels with hair pigmentation genes: a possible hair pigmentation bias

**DOI:** 10.1038/s41598-017-07034-w

**Published:** 2017-08-17

**Authors:** Alexander Neumann, Gerard Noppe, Fan Liu, Manfred Kayser, Frank C. Verhulst, Vincent W. V. Jaddoe, Elisabeth F. C. van Rossum, Henning Tiemeier

**Affiliations:** 1 Department of Child and Adolescent Psychiatry/Psychology, Erasmus MC University Medical Center Rotterdam, Rotterdam, The Netherlands; 2 Department of Pediatrics, Erasmus MC University Medical Center Rotterdam, Rotterdam, The Netherlands; 3 Key Laboratory of Genomic and Precision Medicine, Beijing Institute of Genomics, Chinese Academy of Sciences, Beijing, China; 4 Department of Genetic Identification, Erasmus MC University Medical Center Rotterdam, Rotterdam, The Netherlands; 5 The Generation R Study Group, Erasmus MC University Medical Center Rotterdam, Rotterdam, The Netherlands; 6 Department of Internal Medicine, Erasmus MC University Medical Center Rotterdam, Rotterdam, The Netherlands

## Abstract

Cortisol concentrations in hair are used to create hormone profiles spanning months. This method allows assessment of chronic cortisol exposure, but might be biased by hair pigmentation: dark hair was previously related to higher concentrations. It is unclear whether this association arises from local effects, such as increased hormone extractability, or whether the association represents systemic differences arising from population stratification. We tested the hypothesis that hair pigmentation gene variants are associated with varying cortisol levels independent of genetic ancestry. Hormone concentrations and genotype were measured in 1674 children from the Generation R cohort at age 6. We computed a polygenic score of hair color based on 9 single nucleotide polymorphisms. This score was used to predict hair cortisol concentrations, adjusted for genetic ancestry, sex, age and corticosteroid use. A 1-standard deviation (SD) higher polygenic score (darker hair) was associated with 0.08 SD higher cortisol levels (SE = 0.03, p = 0.002). This suggests that variation in hair cortisol concentrations is partly explained by local hair effects. In multi-ancestry studies this hair pigmentation bias can reduce power and confound results. Researchers should therefore consider adjusting analyses by reported hair color, by polygenic scores, or by both.

## Introduction

In the last decade, studies demonstrated that hair is a useful medium to measure chronic cortisol secretion over a period of 3–6 months^1–4^. Each cm of proximal scalp hair represents ca. 1 month cortisol exposure, which makes the measurement of relatively long-term profiles of cortisol and cortisone, a metabolite and precursor of cortisol, feasible^5^. Hair cortisol assessment is therefore an attractive addition to repeated plasma or saliva measurements.

While hair samples are a compelling method, there is concern that hair color might bias measurements. We reported previously that hair color was associated with cortisol and cortisone levels in the Generation R Study, specifically that higher cortisol levels were found in darker hair^2^. Hair pigmentation might directly affect the potential to extract cortisol from hair and thus measured differences may mirror local effects only. Second, the hair cortisol differences might reflect genetic differences in subpopulations (population stratification). For example, since hair color is strongly linked to genetic ancestry, it might be a marker of genetic variations related to cortisol metabolism or sensitivity^3^. Third, color might be a marker for minority status and the related stress, which would explain higher systemic cortisol levels^1, 2, 4^. Distinguishing between these scenarios is important for observational hair cortisol research, since an association between hair color and hair cortisol might introduce a confounding bias.

To explore the nature of the hair color and hair cortisol association, we investigated whether single nucleotide polymorphisms (SNPs) associated with hair pigmentation are associated with hair cortisol levels in childhood independent of genetic ancestry. For this purpose we selected 16 SNPs from 10 genes included in the HIrisPlex system previously developed to predict hair and eye color from DNA^6, 7^. We created a polygenic score of hair color, which predicts hair lightness/darkness on the basis of these pigmentation SNPs. We computed a genetic score as opposed to solely using reported hair color, because the score allows a continuous assessment of hair pigmentation, is objective, and is not affected by the environment. Such a genetic score potentially represents hair pigmentation more accurately. This way we tested the main hypothesis that a genetic score of hair color is associated with hair cortisol and cortisone levels independent of genetic ancestry in children.

## Methods

### Participants

This study was conducted in Generation R. Generation R is a population-based birth cohort aiming to identify early environmental and genetic determinants of development and health^8, 9^. All parents gave informed consent for their children’s participation. The Generation R Study is conducted in accordance with the World Medical Association Declaration of Helsinki and study protocols have been approved by the Medical Ethics Committee of the Erasmus Medical Center, Rotterdam.

**Figure 1:**
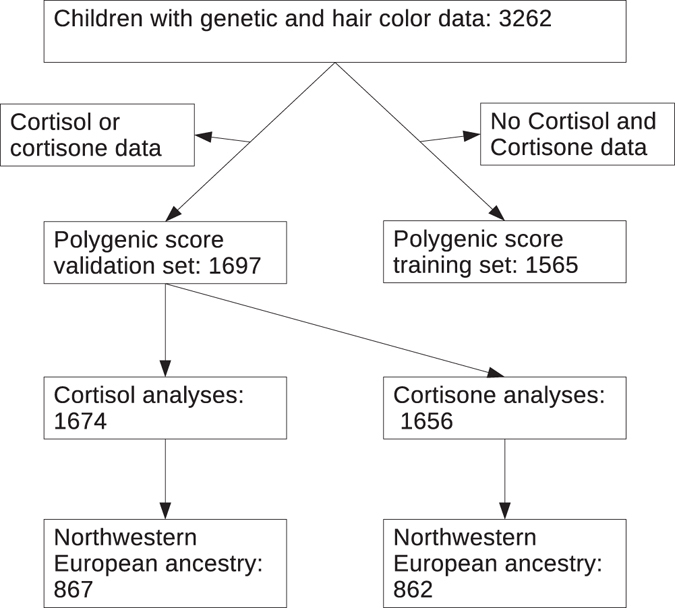
Participant flow chart.

Hair color and genetic information was available in 3262 children. To avoid overfitting of the polygenic score, we split the sample into children with cortisol or cortisone information (n = 1697), the validation sample, and a training sample with neither cortisol nor cortisone (n = 1565) information. Selection and weights of the SNPs for the polygenic pigmentation score were determined in the training set. Hair cortisol or cortisone measurements were available for 1697 children (1674 had cortisol and 1656 had cortisone available). See Fig. 1 for a participant flow chart. Both training and validation samples featured highly admixed populations with a variety of hair phenotypes. See Table 1 for participant characteristics. We additionally studied a subsample of children with genetically northwestern European ancestry to explore whether a hair color bias is present in genetically homogeneous samples. In this sample, 867 measurements of cortisol and 862 of cortisone were available. Finally, we also analyzed subgroups of ethnic minorities grouped by national original of a geographical region: Africa (Africa, Cape Verde, Morocco; n = 193), Asia (Asia, Indonesia; n = 46), Caribbean (Netherlands Antilles, Suriname; n = 156) and Turkey (n = 147).

**Table 1:** Participant Characteristics.

Characteristic	Multi-ancestry			Northwestern European ancestry	
	Training Sample	Cortisol Sample	Cortisone Sample	Cortisol Sample	Cortisone Sample
n	1565	1674	1656	867	862
Cortisol/Cortisone quantiles (in pg/mg)					
25%	—	0.91	5.31	0.69	4.80
50%	—	1.65	7.78	1.28	6.61
75%	—	3.26	12.49	2.71	11.85
Hair Color (in %)					
Sandy red	1	2	2	3	3
Red or Chestnut	1	1	1	2	2
Blond	30	26	26	47	46
Dark Blond	33	26	27	38	39
Brown	13	19	18	9	9
Dark Brown	10	15	15	1	1
Brownish black/Black	13	11	10	0	0
National origin (in %)					
Dutch	65	58	59	90	90
Turkish	6	9	9	0	0
Surinamese	6	7	7	0	0
Other European	8	7	7	6	6
Moroccan	3	7	7	0	0
Cape Verde	2	3	3	0	0
Netherlands Antilles	2	2	2	0	0
African	2	2	2	1	1
American, Non-Western	2	2	2	1	1
Asian, Non-Western	3	2	2	0	0
Indonesian	0	1	1	0	0
Girls (in %)	49	51	52	48	49
Age (mean in years)	6.15	6.19	6.19	6.04	6.04
Corticosteroid use (in %)	—	8	8	9	9

### Genotyping

In Generation R DNA was extracted from whole blood at birth and analyzed using Illumina 610 K/660 W. We filtered for sample (≥97.5%) and SNP call rates (≥95%), minor allele frequency ≥1% and deviations from Hardy-Weinberg equilibrium (p < 10^−7^). Excess heterozygosity, gender accuracy, and relatedness were tested. We used MACH 1.0^10^ to impute to the 1000 Genomes Iv3 reference 11.

We selected 22 SNPs from the HIrisPlex System related to hair color prediction. Nine SNPs were directly genotyped in Generation R and 13 were available as imputed genotypes. Of these, 3 were excluded due to poor imputation quality (R^2^ < 0.3) and 2 due to a minor allele frequency below 1% (Supplementary Table S1, available). SNPs were included as allele dosage in all analyses.

**Figure 2:**
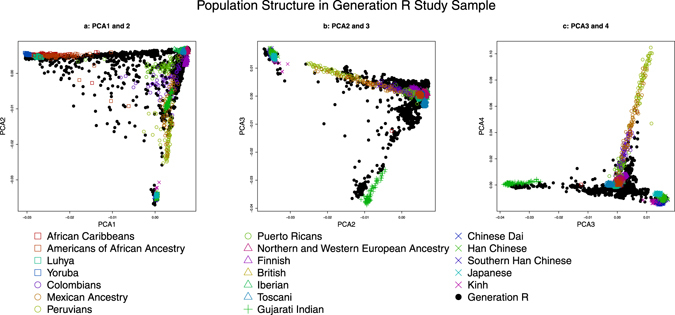
(**a**–**c**) Comparison of genetic ancestry in the Generation R Study sample and the 1000 Genomes phase 3 populations based on the first four principal components of ancestry (PCA). Squares mark African, circles Ad Mixed American, triangles European, crosses South Asian and X indicates East Asian ancestry.

Multidimensional scaling was used for the investigation of genetic ancestry based on the genome-wide SNP data^12^. Twenty principal components of ancestry (PCA) were calculated for the whole Generation R sample (n = 5731) and subsequently used in the subsample of children with available hair color and hormones data, the training and validation samples. Participants exceeding 4 SDs difference with the mean European reference level (HapMap CEU) on any of the first four principal components were classified as non-northwestern European. For analyses restricted to children with northwestern European ancestry, the PCA were recalculated in that Generation R subsample (n = 2830). Again the whole Generation R sample was used for the estimation of PCA. Figure 2 graphically displays the very high population admixture of the training and validation samples, by comparing the genetic ancestry to the 1000 Genomes Phase 3 populations. Supplementary Figure S10 (available) shows correlations between the PCA, SNPs and genetic score.

### Hair Color

Hair color of the children was obtained by parent report and when not available, scored with photographs and videos taken during the research center visit. Inter-coder reliability was calculated with 50 overlapping observations using Krippendorff’s alpha. Alpha was 0.79 between the investigators and 0.69 between the investigators and parents^2^. Hair color was categorized into 7 categories: “sandy red” (1), “red or chestnut” (2), “blond” (3), “dark blond” (4), “brown” (5), “dark brown” (6), “brownish black or black” (7), analyzed as continuous variable (ranging from 1–7) indicating pigmentation intensity. See Table 1 for hair color distribution per sample.

### Hair Cortisol and Cortisone

Cortisol and cortisone concentrations were measured in the proximal three cm scalp hair, as described previously^5^. Briefly, steroids were extracted using LC-grade methanol at 25 °C for 18 h in the presence of deuterium labeled steroids as internal standard. Samples were centrifuged and cleaned using solid phase extraction, after which steroids were quantified by liquid chromatography-tandem mass spectrometry (LC-MS/MS) (Waters XEVO-TQ-S system, Waters Corporation, Milford, MA, USA), using positive electrospray ionization. See Table 1 for hormone concentrations.

### Statistical Analysis

To determine weights for the hair color polygenic score, we first regressed hair color on HIrisPlex SNPs in a single linear model using the training set (n = 1565). Rs16891982 was not included in the training model due to high multicollinearity (Variance Inflation Factor (VIF) = 16.5) caused by strong linkage disequlibrium with rs28777 (r^2^ = 0.92). The model was adjusted for 20 PCA ensuring that only SNPs are selected which have explanatory power beyond being markers for genetic ancestry. Using the regression coefficients of the 9 nominally significant (α = 0.05) SNPs (rs885479, rs1805008, rs1805007, rs28777, rs12896399, rs1042602, rs1393350, rs12821256, rs12203592; see Supplementary Table S2, available) as weights, we calculated a polygenic score in the cortisol/cortisone sample according to β_1_*rs885479 + … + β_9_ * rs12203592. This resulted in a single score indicating the darkness of the hair.

We used the polygenic score to predict cortisol and cortisone in a linear regression model. We included 20 PCA in the main model as covariates (standardized), next to sex, age and corticosteroid use (parent-reported yes/no). Two to ten PCA are commonly recommended to correct for population stratification, depending on the trait and ancestry admixture^13^. A previous Generation R study suggests that the use of four principal components effectively corrects for population stratification in a genome-wide association study of red hair pigmentation. The performance is comparable to adjustment by linear mixed models^12^. Given the strong correlation of hair color with ancestry, we chose to err on the conservative side and included all 20 PCA.

The ancestry corrected analysis using a polygenic score of hair pigmentation was used to test the main hypothesis. However, we performed additional analyses for exploratory and comparative purposes. We also tested individual hair pigmentation SNPs in separate and in a mutually adjusted model. Next, we related observed hair color (treated continuously) to cortisol and cortisone. As the polygenic score was calculated correcting for ancestry, all models were rerun without the additional genetic ancestry adjustments in the regression analyses. Furthermore, we performed sensitivity analyses in the subsample of children of European ancestry as defined by genetic data. We calculated the power of this subsample to detect effect sizes found in the multi-ancestry sample. For this purpose we used the local Cohen’s F^2^ of the fully adjusted pigmentation effect^14^. Finally, we also stratified the main analysis by four ethnic subgroups (African, Asian, Caribbean, Turkish). Given that these classifications are based on national origin rather than genetic data and that the sample sizes are low, we interpret these analyses exploratory.

The hair cortisol and cortisone regression analyses yielded skewed residual distributions. We therefore applied box-cox transformations. The best fitting lambda was −0.26 for cortisol and −0.06 for cortisone, based on the main model. Transformed values were multiplied with −1 to keep directionality. The polygenic score, cortisol and cortisone were standardized to facilitate interpretation.

To investigate potential pleiotropic effects of the hair pigmentation SNPs, i.e. whether the SNPs are associated with hair cortisol via pathways unrelated to hair color, we investigated the heterogeneity of the single SNP estimates as described by Burgess *et al*.^15^. Estimates and standard errors (SE) were extracted for 9 SNPs from ancestry-adjusted models. We meta-analyzed using inverse-variance weighting to calculate Q and I^2^ statistics after orienting the SNP effects. A significant Q or high I^2^ indicate heterogeneity in the effect estimates and can be an indication that the SNP associations are not solely explained by hair color. Heterogeneity is problematic if the pleiotropic effects are in the same direction for a majority of SNPs, which can be detected visually as asymmetry in a funnel plot or by a significant asymmetry test^16^.

Statistical analyses were performed in R 3.3.2^17^. The package MASS 7.3–45^18^ was used for box-cox transformations, metafor 1.9–9^19^ for heterogeneity analyses and funnel plots, pwr^20^ for power analysis, psych 1.6.9^21^ for descriptives and foreign 0.8–67^22^ for reading external files.

## Results

In the training set 9 of the 16 SNPs showed nominally significant (α = 0.05) associations with hair color independent of genetic ancestry as expected (Supplementary Table S2, available). The polygenic score of the nominally significant SNPs explained 35% of the hair color variance in the validation set (n = 1697) (adjusted R^2^). An increase in 1-standard deviation (SD) of the polygenic score was associated with a 0.83-level darker hair (β = 0.83, SE = 0.03, p = 8E-160). For comparison, a polygenic score based on 13 SNPs from a model without genetic ancestry adjustment explained 49% of the variance in this sample.

Hair color, individual hair color SNPs, and the polygenic score of hair color predicted hair cortisol and cortisone levels in models unadjusted for genetic ancestry. These models overestimate the effects due to population stratification and are presented solely for comparison with the main analysis. A 1-level darker hair color was associated with 0.16 SD higher cortisol levels (SE = 0.02, p = 4E-19) and 0.06 SD higher cortisone levels (SE = 0.02, p = 5E-04) (Tables 2 and S3, available). Six hair color SNPs showed independent nominally significant (α = 0.05) associations with cortisol and 2 SNPs did with cortisone. (Tables 3 and S4, available online). A SD higher polygenic score (darker hair) was associated with 0.21 SD higher cortisol levels (SE = 0.02, p = 8E-18) and 0.09 SD higher cortisone levels (SE = 0.02, p = 3E-04) (Tables 2 and S3, available).

**Table 2:** Hair cortisol regressed on hair color and polygenic score of hair color in multi-ancestry sample (n = 1674).

Outcome	Hair Cortisol (standardized)											
Model	Hair Color (no ancestry correction)			Polygenic Score (no ancestry correction)			Hair Color (ancestry correction)			Polygenic Score (ancestry correction)		
Predictor	β	SE	p	β	SE	p	β	SE	p	β	SE	p
Intercept	−0.74	0.24	2E-03	−0.19	0.24	4E-01	0.29	0.27	3E-01	0.33	0.24	2E-01
Hair Color	0.16	0.02	4E-19				0.01	0.03	7E-01			
Polygenic score^1^				0.21	0.02	8E-18				0.08	0.03	2E-03
Sex, female	−0.15	0.05	2E-03	−0.14	0.05	4E-03	−0.19	0.05	7E-05	−0.19	0.05	6E-05
Age, months	0.00	0.00	7E-01	0.00	0.00	3E-01	0.00	0.00	4E-01	0.00	0.00	4E-01
CS use	0.28	0.09	2E-03	0.28	0.09	2E-03	0.32	0.09	2E-04	0.32	0.09	2E-04
PCA1							−0.34	0.04	5E-16	−0.30	0.04	8E-17
PCA2							−0.04	0.03	1E-01	−0.03	0.02	2E-01
PCA3							0.08	0.03	2E-03	0.07	0.02	4E-03
PCA4							0.03	0.02	2E-01	0.03	0.02	2E-01
PCA5							−0.05	0.02	4E-02	−0.05	0.02	3E-02
PCA6							−0.03	0.02	2E-01	−0.03	0.02	2E-01
PCA7							−0.03	0.03	3E-01	−0.03	0.03	3E-01
PCA8							0.00	0.02	1E + 00	0.00	0.02	1E + 00
PCA9							−0.04	0.02	1E-01	−0.04	0.02	9E-02
PCA10							−0.01	0.03	8E-01	0.00	0.03	9E-01
PCA11							−0.02	0.02	4E-01	−0.02	0.02	4E-01
PCA12							−0.03	0.02	3E-01	−0.03	0.02	3E-01
PCA13							−0.02	0.02	3E-01	−0.02	0.02	3E-01
PCA14							−0.05	0.02	3E-02	−0.05	0.02	3E-02
PCA15							0.04	0.02	1E-01	0.04	0.02	1E-01
PCA16							0.03	0.02	3E-01	0.03	0.02	3E-01
PCA17							0.02	0.02	5E-01	0.02	0.02	4E-01
PCA18							0.00	0.02	1E + 00	0.00	0.02	1E + 00
PCA19							−0.05	0.02	4E-02	−0.05	0.02	4E-02
PCA20							0.00	0.02	1E + 00	0.00	0.02	1E + 00

Positive coefficients indicate increases in hormone concentrations. Higher hair color and polygenic scores indicate darker hair. All models were adjusted for sex, age (in months) and corticosteroid (CS) use. Results are shown for models without and with ancestry correction using 20 prinicipal components as covariates (PCA). ^1^Polygenic score is based on 9 SNPs from a training model adjusted for genetic ancestry.

**Table 3:** Hair cortisol regressed on individual pigmentation SNPs in multi-ancestry sample (n = 1674).

Predictor	Seperate models (no ancestry correction)			Mutually Adjusted (no ancestry correction)			Seperate models (ancestry correction)			Mutually Adjusted (ancestry correction)		
	β	SE	p	β	SE	p	β	SE	p	β	SE	p
rs885479	−0.04	0.07	6E-01	0.04	0.07	6E-01	−0.01	0.07	9E-01	−0.02	0.08	8E-01
rs1805008	0.23	0.07	1E-03	0.19	0.07	1E-02	0.14	0.07	5E-02	0.13	0.07	7E-02
rs1805005	0.02	0.08	8E-01	0.01	0.08	9E-01	−0.10	0.08	2E-01	−0.06	0.08	4E-01
rs1805007	0.19	0.09	3E-02	0.11	0.09	2E-01	0.04	0.08	6E-01	0.05	0.09	6E-01
rs2228479	0.03	0.07	6E-01	0.05	0.07	5E-01	0.00	0.07	1E + 00	0.00	0.07	1E+00
rs28777	−0.40	0.05	8E-18	0.02	0.15	9E-01	−0.10	0.06	9E-02	−0.01	0.15	1E+00
rs16891982	0.39	0.04	7E-20	0.31	0.14	3E-02	0.11	0.06	8E-02	0.10	0.15	5E-01
rs2402130	−0.11	0.04	7E-03	−0.02	0.04	7E-01	−0.01	0.04	7E-01	−0.02	0.05	7E-01
rs12896399	0.13	0.03	2E-04	0.03	0.04	4E-01	−0.01	0.04	8E-01	−0.02	0.04	7E-01
rs1042602	0.03	0.04	4E-01	0.01	0.04	7E-01	−0.04	0.04	3E-01	−0.02	0.04	6E-01
rs1393350	0.18	0.04	3E-05	0.11	0.05	2E-02	0.08	0.04	6E-02	0.07	0.05	1E-01
rs12821256	0.19	0.06	2E-03	0.07	0.06	2E-01	0.02	0.06	7E-01	0.02	0.06	7E-01
rs4959270	0.05	0.04	1E-01	0.04	0.03	3E-01	0.01	0.03	8E-01	0.02	0.03	5E-01
rs12203592	−0.07	0.07	3E-01	−0.15	0.07	3E-02	−0.16	0.06	2E-02	−0.17	0.07	1E-02
rs1800407	0.01	0.12	9E-01	0.07	0.11	6E-01	0.04	0.12	7E-01	0.05	0.12	6E-01
rs2378249	0.11	0.05	2E-02	0.13	0.04	5E-03	0.10	0.05	3E-02	0.11	0.05	2E-02
rs683	0.20	0.03	7E-10	0.12	0.03	3E-04	0.05	0.04	1E-01	0.06	0.04	8E-02

SNPs were either included in separate models or mutually adjusted in a single model. Positive coefficients indicate increases in hormone concentrations per effect allele (see Table S1, available).

The polygenic score explained 4.2% of the hair cortisol variance and 0.7% of hair cortisone in a simple regression (genetic ancestry adjusted in training step), whereas hair color explained 4.5% and 0.6% respectively (no genetic ancestry adjustment). For comparison, a polygenic score based on 13 SNPs from a training model without genetic ancestry adjustment explained 5.8% and 0.8% variance. Genetic ancestry explained 8.0% of the cortisol and 3.8% of the cortisone variance. In contrast, national origin (dummy coded) explained 6.1% and 3.5% respectively.

Introducing genetic ancestry into the models substantially decreased the associations. Darker hair color was not associated with hair cortisol (β = 0.01, SE = 0.03, p = 0.70) and cortisone (β = 0.02, SE = 0.03, p = 0.46) (Tables 2 and S3, available). However, 2 hair color SNPs remained nominally significant (α = 0.05) in the cortisol and cortisone models (Tables 3 and S4, available). In the model used for testing the main hypothesis, the polygenic score remained associated with cortisol (β = 0.08, SE = 0.03, p = 2E-03) and cortisone (β = 0.06, SE = 0.03, p = 0.03) (Tables 2 and S3, available). These models showed no substantial multicollinearity (all VIF < 1.46). Restricting the analysis to children with European ancestry changed the coefficients to 0.05 SD for cortisol (SE = 0.03, p = 0.13) and 0.03 SD for cortisone (SE = 0.03, p = 0.39) (Tables S5–S8, available), which were not statistically significant. The cortisol analysis in the European subsample had a power of 59% to detect an association of the same magnitude as found in the multi-ancestry sample (f^2^ = 0.006, power = 86%). Repeating the analysis within ethnic minorities revealed a significant association of the polygenic score with hair cortisol in children of African national origin (β = .22, SE = 0.09, p = 0.01) (Table S9, available). Similar effect sizes in the smaller Caribbean, Asian and Turkish subpopulations did not reach significance.

The associations between 9 single SNPs and hair cortisol showed modest heterogeneity, which was not significant (I^2^ = 43.2%, Q = 13.6, p = 0.09). The funnel plot showed no asymmetry (see Supplementary Figure S11, available) and a regression test was not significant (p = 0.09).

## Discussion

Nine hair color SNPs of the HIrisPlex system explained a large proportion of phenotypic hair color variance in the Generation R Study. The polygenic score of hair color was significantly associated with hair cortisol and cortisone levels after strict adjustment for genetic ancestry. The score itself was based only on SNPs, which showed associations with hair color independent of ancestry. The results suggest that cortisol and cortisone levels found in hair are partly explained by hair pigmentation, and do not represent systemic hormone levels only.

Furthermore, the polygenic score of hair color accounted for the variance in the hair hormone concentrations better than parent-reported/photograph-assessed hair color. While the reported hair color did not show associations independent of genetic ancestry, an independent contribution was found for the genetic markers. This suggests that the predictive value of categorical reporting of hair by parents or researchers is lower than the predictive value of the continuous polygenic score. This may seem surprising, given that the polygenic score explained only part of the reported hair color variance. However, the reported hair color was merely used for weighting and for determining the direction of the SNPs. The additional information on allele dosage and number of pigmentation increasing variants is retained and the initial selection of SNP for the HIrisPlex system was performed in a separate study. It may therefore well be, that the polygenic score is a better representation of hair pigmentation as opposed to the momentary and subjective hair color report. It should be noted that the performance of the presented polygenic score might change in older children or adults. Hair color can change with age, thus it is unclear how predictive the presented score is at other ages, since it is calibrated to school aged children.

An association between hair color and hair cortisol levels had been found previously in dogs^23^, as well as in humans^24^, although not in all studies^25, 26^. The null results in some previous studies could be due to more homogeneous samples compared to this study, which featured a large number of light and dark haired children. The effects of hair pigmentation on hair cortisol were negligible in the European ancestry subsample, in which dark brown and black hair was virtually absent. This suggests that in samples with lower hair color variation and low ancestry variance the hair color bias on cortisol/cortisone measures may be ignored. However, the power in the European sample was also smaller than in the multi-ancestry sample, which may have limited our ability to detect an association. Furthermore, the hair pigmentation bias remained in the non-European subgroups of children stratified by geography.

At present it is unknown what the exact mechanism is underlying the relation between hair pigmentation and cortisol level measures. However, photocrosslinking between the corticosteroid flumethasone and the protein spectrin has been reported^27^. It is conceivable that dark hair is differently affected by UV radiation than light hair and that may also influence a potential crosslinking of cortisol and hair matrix and thereby cortisol extractability.

Whatever the underlying reasons for the observed phenomenon are, these findings have several important implications. In genetically heterogeneous samples (i.e. participants with ancestry from European as well as other non-European regions), hair color certainly adds additional variance to hormonal measurements, which can increase standard errors, and thus adjustment for hair color or genetic markers of hair color could be beneficial. In genetically homogeneous European samples, bias introduced by hair color is small, as shown here, and may be ignored.

A hair color bias could occur in observational cortisol studies with predictors or outcomes, which are associated with hair color. Such population stratification by hair color is conceivable in studies of metabolic traits, psychological stress, and cortisol genetics among others.

In studies of psychological stress in Western multi-ethnic populations for example, the scenario is possible that dark hair is associated with minority status and consequently increased stress exposure. The observed effects of stress on hair cortisol, however, would then be inflated as the association represents the effects of stress on systemic levels as well as those of dark hair pigmentation on hair cortisol levels. In contrast, if light hair is related to higher stress exposure, associations would be deflated. Such studies are typically adjusted for ethnicity, however, given that ethnicity assessments are imperfect and will not be able to account for all hair color biases, further adjustments for hair color are likely useful. Specifically, polygenic scores are beneficial given that their association with hair hormones is partly independent of genetic ancestry. One might even consider adjusting for a polygenic score only instead of ethnicity to reduce chances of overadjustment for true stress effects, though some degree of overadjustment may not be avoidable.

Other research situations, in which hair color might cause misleading results, are future genome-wide association or heritability studies of hair cortisol. These study designs might find genetic effects for hair cortisol, which could be completely driven by hair pigmentation genes and their association with local hair hormone levels. Strategies to counter this phenomenon include the exclusion of hair pigmentation genes before analysis, adjustments for hair cortisol genes in the analysis, or the examination of the linkage disequilibrium between genetic association loci and pigmentation variants after analysis.

We used strict adjustments for genetic ancestry in this study. However, residual confounding by ancestry cannot be completely ruled out and hair pigmentation may remain a marker of ancestry even after controlling for principal components. If this scenario were to explain the observed associations, this study would suggest that adjustments for both principal components and genetic markers of hair color are necessary to correct for population stratification in hair cortisol studies.

The possible implications of this study can be summarized as follows: in genetically heterogeneous study populations hair pigmentation bias can reduce power and lead to confounded associations. Researchers should therefore consider adjusting analyses by (reported) hair color, by polygenic scores or both.

## Electronic supplementary material

Supplementary Tables and Figures
