# Supplementary material for: Predicting hair cortisol levels with hair pigmentation genes: a possible hair pigmentation bias
Source: Sci Rep. 2017 Aug 17;7:8529. doi: 10.1038/s41598-017-07034-w (PMC5561185; doi:10.1038/s41598-017-07034-w)

Predicting hair cortisol levels with hair pigmentation genes: a possible hair pigmentation bias

Alexander Neumann; Gerard Noppe; Fan Liu; Manfred Kayser; Frank C. Verhulst; Vincent W.V. Jaddoe, Elisabeth F.C. van Rossum; Henning Tiemeier

SUPPLEMENTARY TABLES AND FIGURES

Pigmentations SNPs and Hair Color

Table S1: HIrisPlex SNPs available in Generation R.

SNP	Gene	Effect Allele	Other Allele	Effect Allele Frequency	Minor Allele Frequency	Genotyped	R^2
rs1042602	TYR	C	A	0.668	0.332	Yes	1.000
rs1110400*	MC1R	T	C	0.995	0.005	No	0.896
rs11547464*	MC1R	G	A	0.995	0.005	No	0.195
rs12203592	IRF4	C	T	0.935	0.065	Yes	1.000
rs12821256	KITLG	T	C	0.916	0.084	Yes	0.978
rs12896399	SLC24A4	G	T	0.614	0.386	Yes	1.000
rs12913832*	HERC2	A	G	0.570	0.430	No	0.208
rs1393350	TYR	G	A	0.824	0.176	Yes	1.000
rs16891982	SLC45A2	C	G	0.262	0.262	No	0.725
rs1800407	OCA2	C	T	0.954	0.046	No	0.471
rs1805005	MC1R	G	T	0.897	0.103	No	0.460
rs1805006*	MC1R	C	A	0.992	0.008	No	0.193
rs1805007	MC1R	C	T	0.953	0.047	No	0.763
rs1805008	MC1R	C	T	0.929	0.071	No	0.789
rs1805009*	MC1R	G	C	0.992	0.008	No	0.340
rs2228479	MC1R	G	A	0.922	0.078	No	0.910
rs2378249	ASIP	A	G	0.838	0.162	Yes	1.000
rs2402130	SLC24A4	A	G	0.769	0.231	Yes	1.000
rs28777	SLC45A2	A	C	0.774	0.226	No	0.696
rs4959270	EXOC2	C	A	0.582	0.418	Yes	1.000
rs683	TYRP1	C	A	0.474	0.474	No	0.988
rs885479	MC1R	G	A	0.941	0.059	Yes	0.999

R^2 refers to imputation quality.

* indicates SNPs excluded from analysis due to poor imputation quality and/or low minor allele frequency.

Table S2: Hair Color regressed on pigmentation SNPs in multi-ancestry training sample (n=1565).

Predictor	Coefficient	SE	p
(Intercept)	3.18	0.50	3E-10
rs885479	0.16	0.07	3E-02
rs1805008	0.31	0.07	4E-06
rs1805005	0.15	0.08	6E-02
rs1805007	0.61	0.08	4E-13
rs2228479	0.02	0.06	8E-01
rs28777	-0.35	0.07	2E-07
rs2402130	-0.05	0.04	3E-01
rs12896399	0.09	0.04	2E-02
rs1042602	0.09	0.04	2E-02
rs1393350	0.10	0.04	3E-02
rs12821256	0.20	0.05	2E-04
rs4959270	0.02	0.03	5E-01
rs12203592	-0.46	0.06	7E-14
rs1800407	-0.14	0.11	2E-01
rs2378249	0.05	0.04	2E-01
rs683	0.03	0.03	3E-01
PCA1	-15.68	0.88	2E-64
PCA2	-17.90	1.28	7E-42
PCA3	23.81	1.94	5E-33
PCA4	11.43	2.34	1E-06
PCA5	-2.45	3.05	4E-01
PCA6	0.63	3.62	9E-01
PCA7	18.45	3.83	2E-06
PCA8	-16.77	4.39	1E-04
PCA9	6.22	6.23	3E-01
PCA10	1.41	5.43	8E-01
PCA11	-2.61	5.27	6E-01
PCA12	4.90	5.51	4E-01
PCA13	1.49	5.02	8E-01
PCA14	8.07	5.00	1E-01
PCA15	-7.40	4.61	1E-01
PCA16	0.46	4.72	9E-01
PCA17	-8.72	4.67	6E-02
PCA18	5.98	4.63	2E-01
PCA19	10.43	4.72	3E-02
PCA20	-3.76	4.56	4E-01

Regression coefficients indicate increase in 1 level darker hair per number of effect allele.

Hair Cortisone (all ancestries)

Table S3: Hair cortisone regressed on hair color and polygenic score of hair color in multi-ancestry sample (n=1656).

Outcome	Hair Cortisone (standardized)											
Model	Hair Color (no ancestry correction)			Polygenic Score (no ancestry correction)			Hair Color (ancestry correction)			Polygenic Score (ancestry correction)		
Predictor	β	SE	p	β	SE	p	β	SE	p	β	SE	p
Intercept	-0.22	0.24	4E-01	0.01	0.24	1E00	0.07	0.28	8E-01	0.16	0.25	5E-01
Hair Color	0.06	0.02	5E-04				0.02	0.03	5E-01			
Polygenic score[1]				0.09	0.02	3E-04				0.06	0.03	3E-02
Sex, female	-0.22	0.05	1E-05	-0.21	0.05	1E-05	-0.23	0.05	2E-06	-0.23	0.05	2E-06
Age, months	0.00	0.00	8E-01	0.00	0.00	6E-01	0.00	0.00	8E-01	0.00	0.00	9E-01
CS use	-0.09	0.09	3E-01	-0.09	0.09	3E-01	-0.08	0.09	4E-01	-0.08	0.09	4E-01
PCA1							0.05	0.04	2E-01	0.07	0.04	7E-02
PCA2							-0.07	0.03	1E-02	-0.07	0.02	7E-03
PCA3							0.17	0.03	4E-10	0.17	0.03	4E-11
PCA4							-0.03	0.02	2E-01	-0.03	0.02	2E-01
PCA5							-0.06	0.02	1E-02	-0.06	0.02	9E-03
PCA6							-0.06	0.03	3E-02	-0.06	0.03	3E-02
PCA7							-0.02	0.03	4E-01	-0.02	0.03	4E-01
PCA8							-0.03	0.03	2E-01	-0.03	0.03	2E-01
PCA9							-0.03	0.02	2E-01	-0.03	0.02	2E-01
PCA10							0.01	0.03	8E-01	0.01	0.03	7E-01
PCA11							-0.01	0.02	6E-01	-0.01	0.02	5E-01
PCA12							-0.01	0.02	7E-01	-0.01	0.02	7E-01
PCA13							0.00	0.02	1E+00	0.00	0.02	1E+00
PCA14							-0.06	0.03	2E-02	-0.06	0.03	2E-02
PCA15							0.02	0.02	4E-01	0.02	0.02	4E-01
PCA16							-0.03	0.02	2E-01	-0.03	0.02	2E-01
PCA17							-0.01	0.02	6E-01	-0.01	0.02	6E-01
PCA18							0.00	0.02	1E+00	0.00	0.02	1E+00
PCA19							-0.02	0.02	3E-01	-0.02	0.02	3E-01
PCA20							0.02	0.02	5E-01	0.01	0.02	5E-01

Positive coefficients indicate increases in hormone concentrations. Higher hair color and polygenic scores indicate darker hair. All models were adjusted for sex, age (in months) and corticosteroid (CS) use. Results are shown for models without and with ancestry correction (PCA).

[1]Polygenic score is based on 9 SNPs from a training model adjusted for genetic ancestry

Table S4: Hair cortisone regressed on individual pigmentation SNPs in multi-ancestry sample (n=1656).

	Seperate models (no ancestry correction)			Mutually Adjusted (no ancestry correction)			Seperate models (ancestry correction)			Mutually Adjusted (ancestry correction)		
Predictor	β	SE	p	β	SE	p	β	SE	p	β	SE	p
rs885479	-0.01	0.07	9E-01	0.01	0.07	9E-01	0.03	0.07	7E-01	0.05	0.08	5E-01
rs1805008	0.28	0.07	1E-04	0.26	0.07	6E-04	0.26	0.07	3E-04	0.27	0.07	3E-04
rs1805005	-0.08	0.08	3E-01	-0.05	0.08	5E-01	-0.04	0.08	6E-01	0.02	0.09	8E-01
rs1805007	0.10	0.09	3E-01	0.07	0.09	4E-01	0.01	0.09	9E-01	0.05	0.09	6E-01
rs2228479	-0.03	0.07	7E-01	-0.03	0.07	7E-01	-0.05	0.07	5E-01	-0.02	0.07	8E-01
rs28777	-0.13	0.05	8E-03	0.06	0.16	7E-01	-0.04	0.06	5E-01	0.08	0.16	6E-01
rs16891982	0.13	0.04	4E-03	0.11	0.15	4E-01	0.06	0.06	3E-01	0.14	0.16	4E-01
rs2402130	-0.05	0.04	2E-01	-0.01	0.05	9E-01	-0.06	0.04	2E-01	-0.05	0.05	3E-01
rs12896399	0.08	0.03	2E-02	0.05	0.04	2E-01	0.05	0.04	2E-01	0.03	0.04	4E-01
rs1042602	-0.06	0.04	1E-01	-0.06	0.04	1E-01	-0.03	0.04	4E-01	-0.01	0.04	7E-01
rs1393350	0.10	0.04	2E-02	0.06	0.05	2E-01	0.09	0.04	5E-02	0.08	0.05	8E-02
rs12821256	0.10	0.06	9E-02	0.05	0.06	4E-01	0.02	0.06	8E-01	0.01	0.06	9E-01
rs4959270	-0.02	0.04	5E-01	-0.04	0.04	3E-01	-0.03	0.03	4E-01	-0.03	0.04	4E-01
rs12203592	0.03	0.07	7E-01	0.02	0.07	8E-01	-0.01	0.07	9E-01	0.00	0.07	1E+00
rs1800407	-0.16	0.12	2E-01	-0.14	0.12	2E-01	-0.11	0.12	3E-01	-0.10	0.12	4E-01
rs2378249	0.14	0.05	3E-03	0.14	0.05	2E-03	0.12	0.05	1E-02	0.12	0.05	7E-03
rs683	0.06	0.03	6E-02	0.03	0.03	3E-01	0.02	0.04	6E-01	0.03	0.04	5E-01

SNPs were either included in separate models or mutually adjusted in a single model. Positive coefficients indicate increases in hormone concentrations per effect allele (see Table S1).

Cortisol (European Ancestry)

Table S5: Hair cortisol regressed on hair color and polygenic score of hair color in European sample (n=867).

Outcome	Hair Cortisol (standardized, European ancestry)											
Model	Hair Color (no ancestry correction)			Polygenic Score (no ancestry correction)			Hair Color (ancestry correction)			Polygenic Score (ancestry correction)		
Predictor	β	SE	p	β	SE	p	β	SE	p	β	SE	p
Intercept	0.41	0.43	3E-01	0.38	0.41	3E-01	0.42	0.43	3E-01	0.41	0.41	3E-01
Hair Color	-0.01	0.04	8E-01				-0.01	0.04	9E-01			
Polygenic score[1]				0.05	0.03	2E-01				0.05	0.03	1E-01
Sex, female	-0.26	0.07	1E-04	-0.25	0.07	2E-04	-0.28	0.07	6E-05	-0.27	0.07	7E-05
Age, months	0.00	0.01	5E-01	0.00	0.01	5E-01	0.00	0.01	5E-01	0.00	0.01	5E-01
CS use	0.35	0.12	3E-03	0.35	0.12	3E-03	0.35	0.12	3E-03	0.36	0.12	2E-03
PCA1							-0.01	0.03	8E-01	-0.01	0.03	8E-01
PCA2							-0.04	0.05	5E-01	-0.04	0.05	5E-01
PCA3							0.00	0.03	1E+00	0.00	0.03	9E-01
PCA4							0.07	0.03	4E-02	0.07	0.03	5E-02
PCA5							-0.03	0.03	3E-01	-0.03	0.03	3E-01
PCA6							0.03	0.03	4E-01	0.03	0.03	3E-01
PCA7							-0.02	0.04	6E-01	-0.02	0.04	6E-01
PCA8							-0.02	0.04	6E-01	-0.02	0.04	5E-01
PCA9							0.02	0.03	5E-01	0.02	0.03	5E-01
PCA10							-0.05	0.03	1E-01	-0.05	0.03	1E-01
PCA11							0.06	0.04	1E-01	0.05	0.04	1E-01
PCA12							0.04	0.03	2E-01	0.04	0.03	2E-01
PCA13							0.04	0.03	2E-01	0.04	0.03	2E-01
PCA14							-0.01	0.03	7E-01	-0.01	0.03	7E-01
PCA15							0.05	0.03	2E-01	0.05	0.03	2E-01
PCA16							-0.03	0.03	4E-01	-0.03	0.03	3E-01
PCA17							0.02	0.03	5E-01	0.02	0.03	5E-01
PCA18							0.02	0.03	6E-01	0.02	0.03	5E-01
PCA19							-0.01	0.03	9E-01	-0.01	0.03	9E-01
PCA20							0.03	0.03	4E-01	0.03	0.03	4E-01

Positive coefficients indicate increases in hormone concentrations. Higher hair color and polygenic scores indicate darker hair. All models were adjusted for sex, age (in months) and corticosteroid (CS) use. Results are shown for models without and with ancestry correction (PCA).

[1]Polygenic score is based on 9 SNPs from a training model adjusted for genetic ancestry

Table S6: Hair cortisol regressed on individual pigmentation SNPs in European ancestry sample (n=867).

	Seperate models (no ancestry correction)			Mutually Adjusted (no ancestry correction)			Seperate models (ancestry correction)			Mutually Adjusted (ancestry correction)		
Predictor	β	SE	p	β	SE	p	β	SE	p	β	SE	p
rs885479	-0.13	0.11	2E-01	-0.16	0.11	2E-01	-0.13	0.11	2E-01	-0.16	0.11	1E-01
rs1805008	0.14	0.09	1E-01	0.11	0.09	2E-01	0.15	0.09	1E-01	0.12	0.10	2E-01
rs1805005	-0.15	0.11	2E-01	-0.14	0.12	2E-01	-0.18	0.11	9E-02	-0.18	0.12	1E-01
rs1805007	-0.01	0.10	9E-01	0.00	0.11	1E+00	0.00	0.10	1E+00	0.00	0.11	1E+00
rs2228479	0.10	0.09	3E-01	0.07	0.09	5E-01	0.10	0.09	3E-01	0.07	0.09	5E-01
rs28777	-0.12	0.16	5E-01	0.01	0.52	1E+00	-0.13	0.16	4E-01	0.01	0.52	1E+00
rs16891982	0.10	0.15	5E-01	0.11	0.47	8E-01	0.12	0.15	4E-01	0.11	0.47	8E-01
rs2402130	0.00	0.06	9E-01	-0.03	0.07	7E-01	0.00	0.06	1E+00	-0.04	0.07	5E-01
rs12896399	-0.03	0.05	5E-01	-0.04	0.05	4E-01	-0.04	0.05	4E-01	-0.06	0.05	3E-01
rs1042602	-0.05	0.05	4E-01	-0.03	0.06	6E-01	-0.06	0.05	2E-01	-0.04	0.06	5E-01
rs1393350	0.06	0.06	3E-01	0.06	0.06	3E-01	0.08	0.06	1E-01	0.08	0.06	2E-01
rs12821256	-0.07	0.07	3E-01	-0.06	0.07	4E-01	-0.06	0.07	4E-01	-0.06	0.07	4E-01
rs4959270	-0.01	0.05	8E-01	0.01	0.05	8E-01	-0.01	0.05	8E-01	0.01	0.05	8E-01
rs12203592	-0.21	0.08	1E-02	-0.21	0.09	2E-02	-0.21	0.09	2E-02	-0.20	0.09	2E-02
rs1800407	0.08	0.16	6E-01	0.06	0.16	7E-01	0.10	0.17	6E-01	0.08	0.17	6E-01
rs2378249	0.13	0.06	4E-02	0.14	0.06	2E-02	0.13	0.06	4E-02	0.14	0.06	3E-02
rs683	0.03	0.05	6E-01	0.03	0.05	6E-01	0.03	0.05	5E-01	0.03	0.05	6E-01

SNPs were either included in separate models or mutually adjusted in a single model. Positive coefficients indicate increases in hormone concentrations per effect allele (see Table S1).

Cortisone (European Ancestry)

Table S7: Hair cortisone regressed on hair color and polygenic score of hair color in European sample (n=862).

Outcome	Hair Cortisone (standardized, European ancestry)											
Model	Hair Color (no ancestry correction)			Polygenic Score (no ancestry correction)			Hair Color (ancestry correction)			Polygenic Score (ancestry correction)		
Predictor	β	SE	p	β	SE	p	β	SE	p	β	SE	p
Intercept	-0.04	0.43	9E-01	0.09	0.41	8E-01	-0.02	0.43	1E+00	0.12	0.42	8E-01
Hair Color	0.04	0.04	3E-01				0.04	0.04	3E-01			
Polygenic score[1]				0.03	0.03	4E-01				0.03	0.03	4E-01
Sex, female	-0.30	0.07	1E-05	-0.29	0.07	2E-05	-0.31	0.07	9E-06	-0.30	0.07	1E-05
Age, months	0.00	0.01	9E-01	0.00	0.01	9E-01	0.00	0.01	1E+00	0.00	0.01	1E+00
CS use	-0.03	0.12	8E-01	-0.03	0.12	8E-01	-0.03	0.12	8E-01	-0.02	0.12	8E-01
PCA1							0.01	0.03	8E-01	0.01	0.03	8E-01
PCA2							0.04	0.05	4E-01	0.04	0.05	5E-01
PCA3							-0.02	0.03	7E-01	-0.02	0.03	6E-01
PCA4							0.02	0.03	5E-01	0.02	0.03	5E-01
PCA5							0.00	0.03	1E+00	0.00	0.03	9E-01
PCA6							-0.01	0.03	8E-01	-0.01	0.03	8E-01
PCA7							0.00	0.04	9E-01	0.00	0.04	9E-01
PCA8							-0.04	0.04	2E-01	-0.04	0.04	2E-01
PCA9							-0.05	0.03	1E-01	-0.05	0.03	1E-01
PCA10							-0.04	0.03	2E-01	-0.04	0.03	2E-01
PCA11							0.01	0.04	9E-01	0.00	0.04	1E+00
PCA12							0.01	0.03	7E-01	0.01	0.03	7E-01
PCA13							0.03	0.03	4E-01	0.03	0.03	4E-01
PCA14							-0.03	0.03	3E-01	-0.03	0.03	3E-01
PCA15							-0.01	0.03	8E-01	-0.01	0.03	8E-01
PCA16							-0.02	0.03	6E-01	-0.02	0.03	6E-01
PCA17							0.00	0.03	9E-01	0.00	0.03	9E-01
PCA18							0.05	0.03	1E-01	0.05	0.03	1E-01
PCA19							0.02	0.03	6E-01	0.02	0.03	6E-01
PCA20							-0.01	0.03	8E-01	-0.01	0.03	8E-01

Positive coefficients indicate increases in hormone concentrations. Higher hair color and polygenic scores indicate darker hair. All models were adjusted for sex, age (in months) and corticosteroid (CS) use. Results are shown for models without and with ancestry correction (PCA).

[1]Polygenic score is based on 9 SNPs from a training model adjusted for genetic ancestry

Table S8: Hair cortisone regressed on individual pigmentation SNPs in European ancestry sample (n=862).

	Seperate models (no ancestry correction)			Mutually Adjusted (no ancestry correction)			Seperate models (ancestry correction)			Mutually Adjusted (ancestry correction)		
Predictor	β	SE	p	β	SE	p	β	SE	p	β	SE	p
rs885479	-0.06	0.10	6E-01	-0.07	0.11	5E-01	-0.05	0.11	6E-01	-0.07	0.11	6E-01
rs1805008	0.20	0.09	2E-02	0.20	0.09	4E-02	0.21	0.09	2E-02	0.21	0.1	3E-02
rs1805005	-0.09	0.11	4E-01	-0.07	0.12	6E-01	-0.10	0.11	4E-01	-0.07	0.12	5E-01
rs1805007	-0.02	0.10	8E-01	0.00	0.11	1E+00	-0.01	0.1	9E-01	0.02	0.11	8E-01
rs2228479	0.05	0.09	6E-01	0.05	0.09	6E-01	0.06	0.09	5E-01	0.06	0.1	5E-01
rs28777	0.01	0.16	9E-01	0.81	0.52	1E-01	-0.02	0.16	9E-01	0.75	0.53	2E-01
rs16891982	0.06	0.14	7E-01	0.76	0.47	1E-01	0.08	0.15	6E-01	0.73	0.48	1E-01
rs2402130	-0.03	0.06	7E-01	-0.05	0.07	5E-01	-0.03	0.06	6E-01	-0.06	0.07	4E-01
rs12896399	0.00	0.05	9E-01	-0.02	0.05	8E-01	-0.01	0.05	8E-01	-0.03	0.06	6E-01
rs1042602	-0.02	0.05	8E-01	0.02	0.06	7E-01	-0.03	0.05	6E-01	0.01	0.06	8E-01
rs1393350	0.12	0.06	4E-02	0.13	0.06	3E-02	0.13	0.06	3E-02	0.14	0.06	2E-02
rs12821256	-0.03	0.07	7E-01	-0.04	0.07	6E-01	-0.02	0.07	8E-01	-0.03	0.07	7E-01
rs4959270	0.01	0.05	9E-01	0.00	0.05	9E-01	0.00	0.05	1E+00	0.00	0.05	1E+00
rs12203592	-0.01	0.08	9E-01	-0.01	0.09	9E-01	0.00	0.09	1E+00	0.00	0.09	1E+00
rs1800407	0.02	0.17	9E-01	0.00	0.17	1E+00	0.01	0.17	9E-01	0.00	0.17	1E+00
rs2378249	0.11	0.06	7E-02	0.12	0.06	5E-02	0.11	0.06	8E-02	0.12	0.06	6E-02
rs683	0.02	0.05	6E-01	0.02	0.05	7E-01	0.02	0.05	7E-01	0.02	0.05	7E-01

SNPs were either included in seperate models or mutually adjusted in a single model. Positive coefficients indicate increases in hormone concentrations per effect allele (see Table S1).

Table S9: Hair cortisol and cortisone regressed on polygenic score of hair color stratified by national origin of ethnic minorities

National Origin	n	β	SE	p
Cortisol				
Africa	193	0.22	0.09	1E-02
Asia	46	0.29	0.27	3E-01
Caribbean	156	0.08	0.11	5E-01
Turkey	147	0.17	0.10	8E-02
Cortisone				
Africa	185	0.15	0.09	8E-02
Asia	43	0.34	0.22	1E-01
Caribbean	153	0.24	0.15	1E-01
Turkey	141	0.09	0.11	4E-01

Positive coefficients indicate increases in hormone concentrations. Higher polygenic scores indicate darker hair. All models were adjusted for sex, age (in months), corticosteroid use and genetic ancestry.

Correlations between PCA, SNPs and Genetic Score

Figure S10: Pearson correlations between PCA, SNPs and genetic score

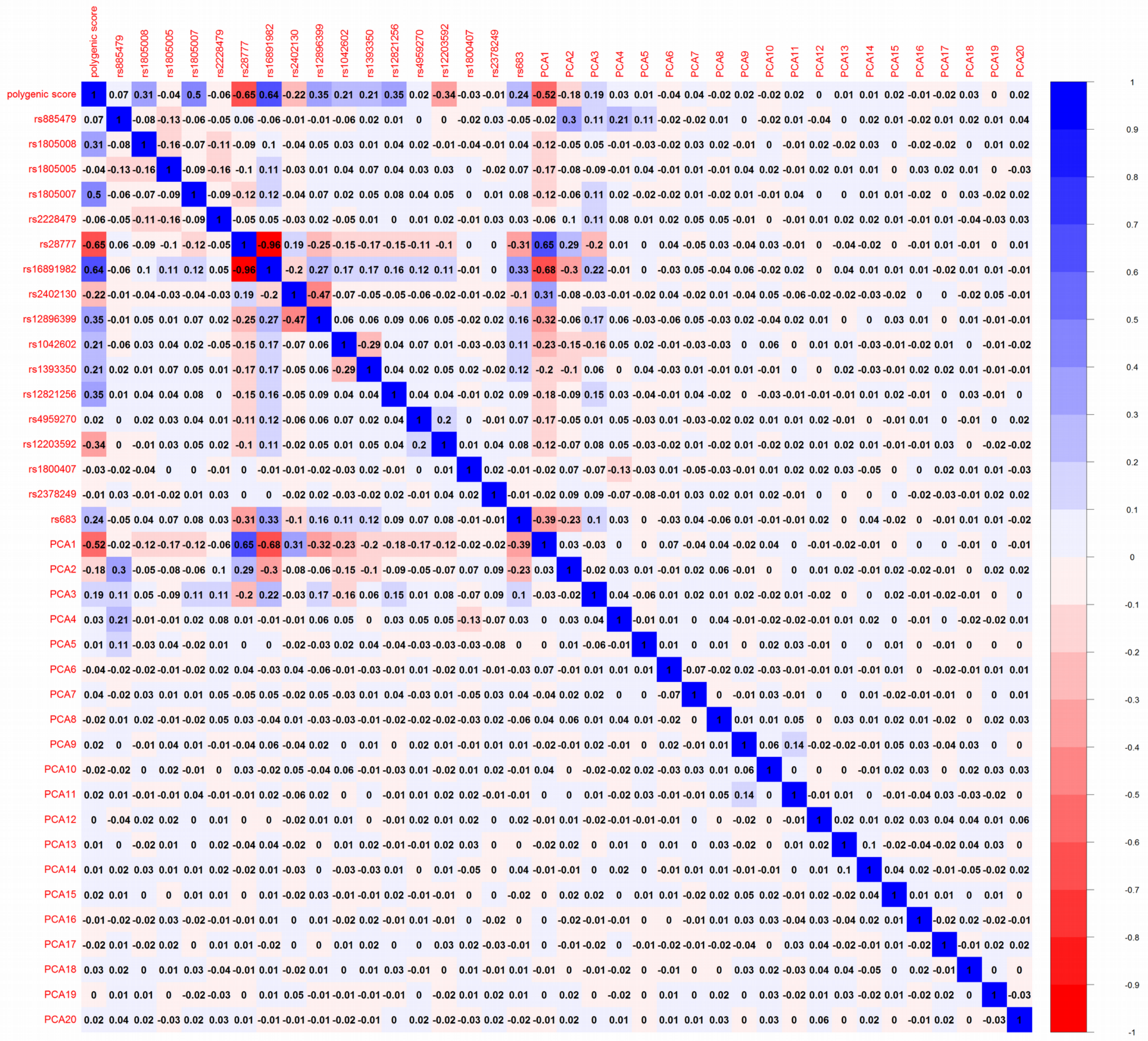

Funnel plot of single SNP associations with hair cortisol

Figure S11: Funnel plot showing standardized estimates of 9 SNPs associated with hair cortisol against their standard error. White area indicates 95% confidence interval. SNPs not included in polygenic score were omitted. Each model was adjusted for sex, age (in months), corticosteroid (CS) use and genetic ancestry (PCA).

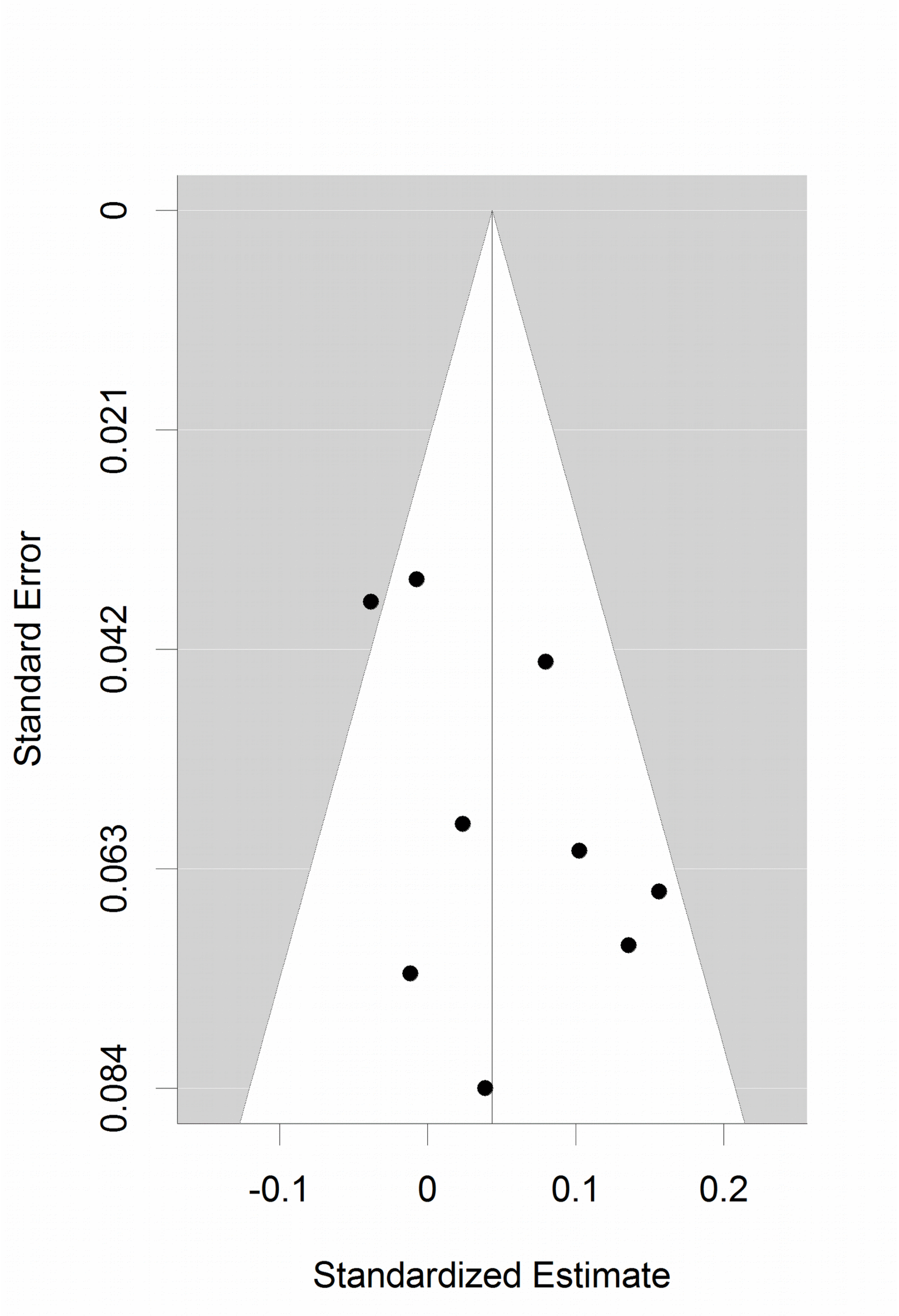

Supplement: Supplementary file 1 — Supplementary Tables and Figures [file 41598_2017_7034_MOESM1_ESM.pdf]
